# Methods and matrices: approaches to identifying miRNAs for Nasopharyngeal carcinoma

**DOI:** 10.1186/1479-5876-12-3

**Published:** 2014-01-06

**Authors:** Jordan L Plieskatt, Gabriel Rinaldi, Yanjung Feng, Paul H Levine, Samantha Easley, Elizabeth Martinez, Salman Hashmi, Nader Sadeghi, Paul J Brindley, Jeffrey M Bethony, Jason P Mulvenna

**Affiliations:** 1 Department of Microbiology, Immunology and Tropical Medicine, School of Medicine and Health Science, George Washington University, Washington, DC, USA; 2 Research Center for Neglected Diseases of Poverty, School of Medicine and Health Science, George Washington University, Washington, DC, USA; 3 Departamento de Genética, Facultad de Medicina, Universidad de la República, (UDELAR), Montevideo 11800, Uruguay; 4 Department of Epidemiology and Biostatistics, The George Washington University School of Public Health and Health Services, Washington, DC 20037, USA; 5 Department of Pathology, School of Medicine and Health Science, George Washington University, Washington, DC, USA; 6 Medical Faculty Associates, The George Washington University, Washington, DC 20037, USA; 7 Infectious Disease and Cancer, Queensland Institute for Medical Research, Brisbane, Australia

**Keywords:** Nasopharyngeal carcinoma, Methods, Biomarker, MicroRNAs, qPCR, Next generation sequencing, RNA-Seq, Tumor

## Abstract

**Background:**

Nasopharyngeal carcinoma (NPC) is a solid tumor of the head and neck. Multimodal therapy is highly effective when NPC is detected early. However, due to the location of the tumor and the absence of clinical signs, early detection is difficult, making a biomarker for the early detection of NPC a priority. The dysregulation of small non-coding RNAs (miRNAs) during carcinogenesis is the focus of much current biomarker research. Herein, we examine several miRNA discovery methods using two sample matrices to identify circulating miRNAs (c-miRNAs) associated with NPC.

**Methods:**

We tested two miRNA discovery workflows on two sample sources for miRNAs associated with NPC. In the first workflow, we assumed that NPC tumor tissue would be enriched for miRNAs, so we compared miRNA expression in FFPE from NPC cases and controls using microarray and RNA-Seq technologies. Candidate miRNAs from both technologies were verified by qPCR in FFPE and sera from an independent NPC sample set. In a second workflow, we directly interrogated NPC case and control sera by RNA-Seq for c-miRNAs associated with NPC, with candidate c-miRNAs verified by qPCR in the sera from the same independent NPC sample set.

**Results:**

Both microarray and RNA-Seq narrowed the miRNA signature to 1-5% of the known mature human miRNAs. Moreover, these two methods produced similar results when applied to the same sample type (FFPE), with RNA-Seq additionally indicating “unknown” miRNAs associated with NPC. However, we found different miRNA profiles in NPC sera compared to FFPE using RNA-Seq, with the few overlapping miRNAs found to be significantly up-regulated in FFPE significantly down-regulated in sera (and vice versa). Despite the different miRNA profiles found in FFPE and sera, both profiles strongly associated with NPC, providing two potential sources for biomarker signatures for NPC.

**Conclusions:**

We determined that the direct interrogation of sera by RNA-Seq was the most informative method for identifying a c-miRNA signature associated with NPC. We also showed that there are different miRNA expression profiles associated with NPC for tumor tissue and sera. These results reflect on the methods and meaning of miRNA biomarkers for NPC in tissue and peripheral blood.

## Background

Nasopharyngeal carcinoma (NPC) is an Epstein Barr virus (EBV) associated squamous cell carcinoma of the head and neck. While notable for its distinct geographical distribution [1,2], this solid tumor is also remarkable for its extensive interaction with the tumor microenvironment and the host immune system (e.g., immunoediting) [3]. Peripheral blood and saliva collected from NPC patients often contains numerous tumor-derived products, including cytokines [4,5], non-cytokine tumor proteins [4,6-10], and viral nucleic acids, as well as EBV antibodies and antigens [3,11-15]. These circulating tumor and oncogenic viral products represent an easily accessible source for biomarkers and make NPC, as Gourzones et al. [3] state, a “privileged model” for peripheral blood biomarkers.

In this manuscript, we focus on methods that could be used to identify circulating miRNA (c-miRNAs) biomarkers. These small non-coding RNAs are key players in post-transcriptional expression regulation and are involved in a wide range of cellular processes, often circulating as long-range signaling molecules in the peripheral blood [16-20]. Numerous miRNAs have been found in nearly all sample matrices associated with cancer, including tumor tissue, sera, plasma, and saliva. Moreover, it has been demonstrated that miRNA levels are “stable, reproducible, and consistent among individuals with the same cancer” [21], and are being used as biomarkers for breast [22], colorectal [23] and ovarian cancers [24]. When compared to other biomarker species, miRNAs offer unique advantages: (1) they can be amplified using qPCR, enabling their levels to be verified and quantified with a high degree of sensitivity and specificity in serum or plasma; (2) multiple miRNAs can be amplified by multiplex qPCR, which (3) enables the simultaneous detection of dysregulated miRNAs (miRNA signatures) within the same sample. In addition, high quality small RNA preparations, enriched with miRNAs, can be extracted from formalin-fixed paraffin embedded (FFPE) tissue [25-28], the clinical standard for the processing NPC tumor samples, enabling us to utilize our extensive repository of NPC biospecimens from around the world [29-34].

**Figure 1:**
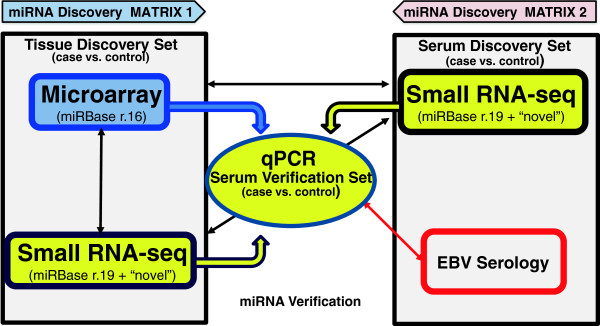
**Flow diagram of methods and matrices explored for discovering a circulating miRNA signature for nasopharyngeal carcinoma in a training set of samples.** Both targeted, (microarray) and an untargeted (small RNA-Seq) approaches were used to determine relative expression levels of miRNA in FFPE NPC tumor tissue compared to normal control tissue (black arrows). Based on this analysis, qPCR was used to confirm miRNA expression levels in tissue and to target these miRNAs in sera (colored thick arrows). qPCR results showed miRNA expression levels in sera had little correlation with tissue RNA-Seq used to profile expression levels in this same sample matrix. Cognate serology support verification and correlation of detected miRNAs (red arrow).

Herein, we assess two methods (microarray and RNA-Seq) for miRNA expression profiling applied to two different sample types (FFPE and sera) from NPC cases and age, sex, and geographically matched controls. While sera presents the richest and most easily accessible source for circulating miRNA biomarkers, the dynamic range and low abundance of most biomarker species in sera makes it a challenging matrix for initial miRNA biomarker discovery. As with other studies of solid tumor biomarkers [20,35,36], our workflow assumed that abundant miRNAs from the primary tumor enter into the bloodstream, where they can be utilized as biomarkers, as shown for breast, lung and prostate cancers [37,38]. Accordingly, we assessed two methods for miRNA biomarker discovery based on sample type and discovery platform (Figure 1). In the first biomarker discovery workflow, we started with the interrogation of FFPE from confirmed NPC cases versus matched healthy controls using “targeted” and an “untargeted” discovery platforms, i.e., microarray versus RNA-Seq, respectively. Subsequently, a set of candidate miRNAs associated with NPC was verified using qPCR for their detection and quantitation in sera. This method was based on the assumption that NPC, more than any other solid tumor, has an extensive interaction with the host, especially the host immune system (i.e., “immunoediting”) and tumor micro-environment [3]. In the second biomarker discovery workflow, we directly interrogated NPC case and control sera by RNA-Seq for circulating miRNAs (c-miRNAs) associated with NPC, with candidate c-miRNAs verified in serum by qPCR. While most studies of miRNA expression in cancer have focused on biomarker discovery in either tumor tissue or sera/plasma, this study is among the first to compare the different methods and matrices for biomarker discovery for NPC.

## Methods

### Sample characteristics and preparation

#### Case and control tissue preparation

Detailed characteristics of the FFPE samples used in this study are shown in Tables 1 and 2. In brief, formalin fixed paraffin-embedded tissue blocks from four cases of histologically confirmed non-keratinizing undifferentiated NPC (the most common form of NPC) diagnosed between 2004 and 2012 and four samples of non-neoplastic nasorespiratory tissue were obtained from the biological repository at the Department of Pathology, The George Washington University Hospital, Washington, DC. Tissue specimens were fixed in 10% neutral buffered formalin and processed into paraffin wax (FFPE) by routine methods. Two of the control samples were non-neoplastic sinusoidal mucosal tissue biopsied from ipsilateral sites distal to the primary tumor (at an undetermined distance) at the time of the original diagnostic procedure. The other control tissues were chronic allergic sinusitis, chronic rhinosinusitis, sinus mucosa with chronic inflammation, and sinus mucosa with no significant histologic abnormality. All tissue sections were reviewed independently by two pathologists (E.M. and S.E.E.) to confirm the histopathologic diagnosis and to classify tumors by World Health Organization (WHO) terminology [35]. The age, sex, ethnicity, Tumor Node and Stage (TNM) [39], and WHO classification of nasopharyngeal [40] tumors are described in detail in Table 1. The cases included three men and one woman between 46 and 80 years of age. One master re-cut hematoxylin and eosin (H&E) stained slide was made for samples. For the NPC cases, paraffin blocks containing >90% viable tumor were selected for dissection. Two FFPE samples of non-keratinizing differentiated and two of non-keratinizing undifferentiated NPC as well as 8 non-neoplastic control samples (8 samples) underwent RNA isolation and microarray analysis as described below. Due to limited tissue area and recovered RNA, RNA sequencing was performed on only three of the four tumor samples (Tumor 2 omitted) and only two of the four non-neoplastic nasorespiratory tissue samples. Representative images of NPC cases and non-neoplastic controls are shown in Additional file 1.

**Table 1:** Describes the formalin fixed paraffin embedded (FFPE) tissue from biopsies of histologically confirmed nasopharyngeal carcinoma tissue and two corresponding controls from non-tumor tissue and two biopsies of patients with nasal polyposis from the Department of Pathology, George Washington University, DC, USA

**Sample**	**Histologic type**	**WHO classification**^**1**^	**TNM**^**2**^	**Sex**	**Age**	**Ethnicity**	**Origin**	**History at presentation**
**Tumor FFPE**								
Tumor 1	Differentiated	Non-keratinizing	T4N2M0	M	46	Asian	USA	Right sphenoid mass; Right neck mass; bloody nasal discharge; facial numbness; headache
Tumor 2	Differentiated	Non-keratinizing	T3N0M0	F	80	Hispanic	USA	Right sphenoid mass; Right neck mass; bloody nasal discharge; facial numbness; headache
Tumor 3	Undifferentiated	Non-keratinizing	T4N2M0	M	47	Asian	USA	Large mass involving posterior wall of nasopharynx with superior extension and inferior involvement right oropharyngeal wall
Tumor 4	Undifferentiated	Non-keratinizing	T3N1M0	M	49	NA	USA	Right sided 3 cm neck mass; hypermetabolic activity in right nasopharynx on PET/CT
**Control FFPE**								
Control 1	Non-neoplastic tissue adjacent to Tumor 1					Asian	USA	Sinonasal mucosa adjacent to NPC tumor
Control 2	Non-neoplastic tissue adjacent to Tumor 2					Hispanic	USA	Sinonasal mucosa adjacent to NPC tumor
Control 3	--	--	--	F	43	African-American	USA	Nasal polyposis with chronic rhinosinusitis; large nasopharyngeal mass
Control 4	--	--	--	M	65	NA	USA	Nasal polyposis with chronic rhinosinusitis; large nasopharyngeal mass

FFPE are presented by histological type, WHO classification [40], TNM staging [39], sex, age, ethnicity, origin of FFPE, and the history at presentation. Currently, the WHO divides NPC into keratinizing, non-keratinizing, and basaloid squamous cell carcinoma. Non-keratinizing NPC, which is the most common form of NPC, is further subdivided into differentiated and undifferentiated.

^1^[40]; ^2^[39]; NA indicates that data were not available.

**Table 2:** Shows the serum samples for this study consists of anonymously coded vials of sera from histopathologically confirmed cases of nasopharyngeal carcinoma (NPC) and their corresponding healthy controls from studies undertaken by the National Cancer Institute, National Institutes of health, USA and detailed in manuscripts 30, 31, 32, and 40

**Origin**^**1**^	**Sample**	**Sex**			**Age**	**WHO **[40]** classification**	**Subtype**	**Ethnicity**	**Ref**
		**N**	**M**	**F**	**Mean (95% CI)**				
Germany^4^	Case^2^	10	10	0	60.5 (50.7, 70.3)	Non-keratinizing	Differentiated	Caucasian	[31]
	Control	6	5	1	NA^3^	Healthy		Caucasian	
USA^5^	Case	5	3	2	60.0 (48.9, 71.2)	Non-keratinizing	Differentiated	Caucasian	[32]
	Control	3	2	1	61.0 (41.2, 80.8)	Healthy		Caucasian	
Malaysia^6^	Case	12	12	0	49.5 (44.9, 54.1)	Non-keratinizing	Undifferentiated	Asian	[29,41]
	Control	4	4	0	50.0 (39.6, 60.4)	Healthy		Asian	
Total	Case	27	25	2	52.1 (47.8, 56.3)	--	--	--	--
	Control	13	11	2	53.7 (43.8, 64.1)	--	--	--	

The samples were maintained under Good Biobanking Practices at the Biorepositories and Biospecimen Research Branch (BBRB), National Cancer Institute, Frederick, MD, National Institutes of Health, USA.

^1^All specimens biobanked and received from the Biorepositories and Biospecimen Research Branch (BBRB), National Cancer Institute, National Institute of Health, USA.

^2^The term “case” refers to histologically confirmed nasopharyngeal carcinoma.

^3^NA indicates an absence of data for this group.

^4^Ear Nose and Throat Clinic, Cologne University, Germany.

^5^North American NPC Study, Mayo Clinic, USA.

^6^Institute of Radiotherapy, Oncology and Nuclear Medicine at the General Hospital, Kuala Lumpur, Malaysia.

#### Case and control sera

Detailed characteristics of the serum samples used in this study are shown in Table 2 and in manuscripts [29-34]. In brief, serum samples for this study consisted of anonymously coded vials of sera from histopathologically confirmed cases of nasopharyngeal carcinoma (NPC) and their corresponding healthy controls from studies undertaken by the National Cancer Institute (NCI), National Institutes of Health (NIH), USA, as part of a multicenter studies involving institutions in the USA, Germany, and Malaysia [29-31,33,34] and maintained and shipped from the Biorepositories and Biospecimen Research Branch (BBRB), of the NCI-NIH, Frederick, MD, USA. As a part of these NCI studies, sera were matched for age, ethnicity, sex, and country of residence with sera from healthy controls. For this study, 16 serum samples were from a Malaysian collection, which were shipped from a treatment facility in Kuala Lumpur, Malaysia to the National Cancer Institute, Bethesda, MD [32]. Twenty-four samples were from a multicenter study that included samples from ENT Clinic at Cologne University, Germany [31], and from the Massachusetts Eye and Ear Infirmary at the Massachusetts General Hospital in Boston [29]. All sera were from patients who underwent complete clinical investigation to determine TNM status (see manuscripts).

### Ethical approval

The GWU IRB determined that the study samples used in this study did not meet the definition of human subjects research; i.e., a living individual about whom an investigator conducting research obtains: a) data through intervention or interaction with the individual or b) private identifiable information. This determination was made since the samples were limited to preexisting, de-identified specimen analysis labeled with a random code (see attached PDF).

### Isolation of RNA

#### FFPE

Total RNA was isolated from FFPE sections using the miRNeasy FFPE kit (Qiagen) according to manufacturer’s protocol. Briefly, 320 μL Deparaffinization Solution (Qiagen) was added followed by brief vortexing, centrifugation and incubation for 3 minutes at 56°C. Buffer PKD was added to the samples before centrifugation and proteinase K treatment at 56°C for 15 minutes. The samples were then incubated at 80°C for 15 minutes to partially reverse formaldehyde modification. The lower phase was then transferred to a new tube and DNase digestion was performed at room temperature for 15 minutes. 500 μL RBC buffer and 100% ethanol (Acros Chemical) were added to the samples and transferred to the RNeasy MiniElute column. The column was washed twice with RPE, and RNA eluted in 30 μL RNase-free water.

#### Sera

miRNAs were isolated from sera using the QIAamp Circulating Nucleic Acid Kit (Qiagen) according to the manufacturer’s protocol for purification of circulating miRNAs from serum, plasma or urine. Up to 0.5 mL of serum was digested with 400 μL proteinase K. Buffer ACL, without carrier RNA and buffer ATL was then added and the sample was pulse-vortexed for 30 seconds before incubation at 60°C for 30 min. Buffer ACB and isopropanol (Acros) were added to the sample and incubated for 5 minutes on ice. The samples were applied to the QIAamp Mini column using the QIAvac 24 Plus. The columns were washed with buffer ACW1, ACW2 and ethanol, dried at 56°C for 5 minutes and miRNAs eluted in 50 μL Buffer AVE. Concentration, purity and integrity (RIN) for the RNA were determined by spectrophotometry (Nanodrop 1000) and Agilent 2100 Bioanalyzer using the Agilent RNA 6000 Nano, pico, and Small RNA kits as appropriate. RNAs were stored at < −50°C.

### Microarray analysis

Total RNA (250 μg) isolated from each FFPE case was labeled with Cyanine 3-pCp using Agilent miRNA labeling and hybridization kits, hybridized to the Agilent human miRNA microarray (miRBase Release 16.0), and scanned. The feature intensities were transferred to digital data and Log2 transformed using Feature Extraction (V.10.7). For data analysis, inter-sample variance was normalized using quantile normalization strategies. Hierarchical clustering by Euclidean distance was used to cluster samples and groups with similar miRNA profiles. Differential analysis was performed using an unpaired *t*-test, ANOVA, and fold-change analysis.

### Small RNA sequencing

#### Rio-Zero pretreatment of total RNA from FFPE

RNA purified from FFPE were depleted of rRNA by treatment with the Ribo-Zero rRNA Removal Kit (Cat. No. RZH1086, Epicentre), as described by the manufacturer. Briefly, biotinylated capture probes directed against rRNA sequences were added to total RNA samples and allowed to hybridize. Biotinylated complexes were removed using streptavidin-conjugated microbeads and non-ribosomal RNAs precipitated in ethanol.

#### Library preparation and sequencing

Libraries were prepared for small RNA sequencing using the TruSeq Small RNA Sample Prep Kit (Illumina). Illumina libraries were constructed from 1,000 ng of total RNA. Briefly, indexed oligonucleotide adapters were ligated to both the 3′-hydroxyl end and the 5′-phosphate end of the miRNAs using T4 RNA Ligase (New England Biolabs). RNA was reverse-transcribed and amplified using 14 cycles of PCR with primers targeting the 5′ and 3′ adapters, a specific index sequence, and Illumina sequencing adapters. The resulting products were analyzed and quantified using Agilent 2100 BioAnalyzer and the molar amount of mature miRNA present in the library was estimated by integrating the area under the curve in the 145 – 160 bp range. Individual libraries were mixed to create multiplexed pools, the mixture was gel purified, and the 145–160 bp range of RNA excised from the gel, crushed using a Gel Breaker tube (IST Engineering), eluted with nuclease-free water, and precipitated in ethanol. The concentration of the final library pool was determined using the PicoGreen system (Invitrogen) and the size distribution of the pool by the Agilent 2100 BioAnalyzer. Library pools were normalized to 2 nM for sequencing. Sequencing was performed using an Illumina Genome Analyzer IIx. Library preparation and small RNA sequencing was performed by Expression Analysis, A Quintiles Company (Durham, NC).

### Micro-RNA alignment, mapping and annotation

Adapter sequences were clipped from deep sequencing reads using FastqMcf (http://code.google.com/p/ea-utils/wiki/FastqMcf) and initial quality assessment performed using FastQC (http://www.bioinformatics.babraham.ac.uk/projects/fastqc/). To analyze miRNA expression profiles both miRDeep 2.0.0.5 [42] and miRExpress 2.0 [43] were used. Briefly, short reads were mapped to the human (UCSC hg19) and the Human herpes virus 4 (Epstein-Barr virus) genome (NCBI NC_007605.1) allowing a minimum read length of 18, zero mismatches in the seed region and a maximum of five genomic loci. Known human and EBV miRNAs were identified and quantified based on miRBase Release 19 [44] entries. Using miRExpress known human and EBV miRNAs were identified from miRBase Release 19 with an alignment identity of 1% a tolerance range of four and a similarity threshold of 0.8 in the analysis. Differential expression analysis was performed separately for miRDeep and miRExpress using a negative binomial distribution in EdgeR [45]. Only miRNAs with at least one count per million in at least two samples were used in expression analysis and counts were normalized using the trimmed mean of M-values normalization method [46]. The analysis was performed using moderated tagwise dispersions. Differentially expressed miRNAs were defined as having a Benjamini and Hochberg corrected *p* value of ≤ 0.05.

### Quantitative real time PCR (qPCR)

cDNA was generated from 32–125 ng RNA using the miScript RT II kit (Qiagen) and the qPCR was performed using the miScript SYBR Green PCR Kit (Qiagen) on custom printed 96 well miScript miRNA arrays (SABiosciences). Selected miRNAs and normalization controls printed on the plate are shown in Additional file 2. The qPCRs were performed using a BioRad iCycler iQ5 with an initial activation step of 95°C for 15 minutes followed by 40 cycles of 3-step cycling (denaturation, 15 sec, 94°C; annealing, 30 sec, 55°C; and extension, 30 sec, 70°C) followed by a melting curve analysis for 81 cycles at 55°C and 20 sec dwell time. C_t_ values were exported and analyzed using SABiosciences tool (http://pcrdataanalysis.sabiosciences.com/mirna) and relative quantitation was performed using the ΔΔC_t_ method [47]. SNORD and RT controls were utilized for normalization of samples.

### Database accession

RNA sequence data have been submitted to the Sequence Read Archive (SRA, National Center for Biotechnology Information, U.S. National Library of Medicine, Bethesda, MD) under accession number SRP029599. Microarray data were prepared according to MIAME standards and deposited in the GEO (Gene Expression Omnibus Database, National Center for Biotechnology Information, U.S. National Library of Medicine, Bethesda, MD) under accession number GSE46172.

## Results

### FFPE tissue yielded RNA of sufficient quality for downstream analysis

Using the Qiagen miRNeasy FFPE kit, starting material of 2 × 10 μm sections provided RNA yields of ~100 ng/μm. The purified RNA exhibited 260/280 and 260/230 ratios of ~2.0 and ~1.9, respectively, which is considered an acceptable level of purity for the downstream applications in our program, including RNA-Seq. Both electrophoresis, using TBE-urea gels, and analysis with the Agilent 2100 BioAnalyzer (not shown) were used to monitor RNA profiles. Electropherograms of RNA isolated from FFPE showed broad peaks at < 100 nt, which indicated that the sample included small RNA species (not shown). The integrity (or RIN score) of the samples ranged between 2–3. When taken with the absence of 28S and 18S ribosomal RNA peaks, this suggested the degradation of larger RNA species. However, given the robustness of miRNAs in FFPE tissue [48] and reports from other groups [49] that RIN values have negligible effect on miRNA results, the purified RNA was considered suitable for further analysis.

Microarray and RNA-Seq exhibited similar miRNAexpression profiles in FFPE tissue.

**Figure 2:**
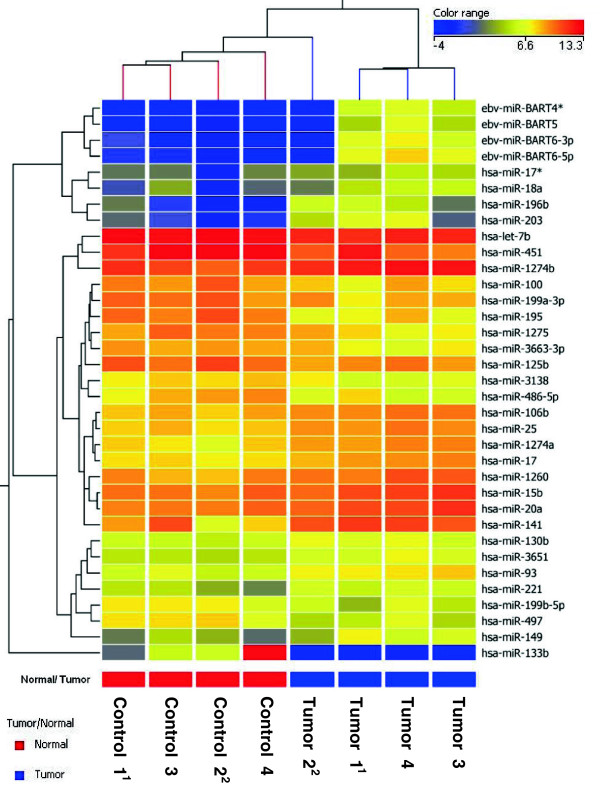
**Hierarchical clustering of significantly changed miRNAs in NPC versus normal control tissue.** Hierarchy heatmap generated using a Euclidean distance metrics and centroid linkage method. Expression levels of each miRNA in each samples are represented by different colors signifying hybridization intensities. Superscript numbering on sample names denotes those from the same individual (paired NPC/control tissue). Further analysis with statistical information is presented in Additional file 3.

High-throughput analysis of miRNA expression profiles typically utilizes small RNA microarrays (i.e. targeted approach) or RNA-Seq (i.e., untargeted approach) [20]. To compare the utility of the two techniques for biomarker discovery, both approaches were used to profile miRNA expression in NPC FFPE tissue compared to non-neoplastic nasorespiratory FFPE control tissue. For microarray analysis, 250 ng purified RNA from eight FFPE samples (four NPC FFPE and four non-neoplastic nasorespiratory tissue FFPE) were analyzed using the Agilent human miRNA microarray (miRBase Release 16.0), which includes 1,205 human and 144 viral miRNA targets. After hierarchical clustering and statistical analysis of differential expression, 31 miRNAs (13 down-regulated and 18 up-regulated) exhibited a fold change (FC) greater than two (*p* < 0.05) in tumor tissue icompared to non-neoplastic nasorespiratory control tissue (Figure 2). Four EBV miRNAs, (Bart4*, Bart5, Bart6-3p and Bart6-5p) were significantly up-regulated in the four FFPE from the histologically confirmed NPC cases versus the non-neoplastic nasorespiratory tissue controls. Absolute fold changes along with *p*-values for all dysregulated miRNAs obtained via microarray are shown in Additional file 3.

**Figure 3:**
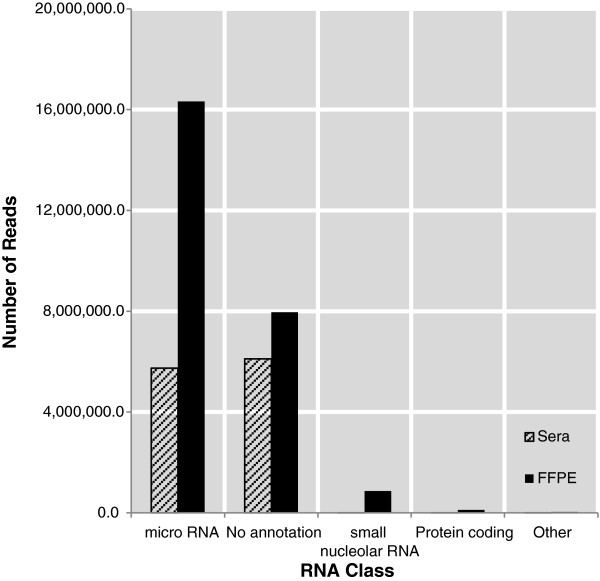
**Mapping of small RNA reads from FFPE and sera samples.** Average reads and mapping per sample (total of five FFPE and four sera). > 25 million reads were achieved for FFPE of which >16 million were miRNAs. ~ 12 million reads in total were sequenced from the sera, of which ~ 6 million were mapped to miRNAs.

For RNA-Seq, one μg RNA from five FFPE tissue samples (3 NPC FFPE and 2 non-neoplastic nasorespiratory tissue FFPE) was sequenced using the Illumina platform. (Two non-neoplastic nasorespiratory tissue FFPE (Control 2 and Control 4) and one NPC FFPE (Tumor) were not sequenced due to insufficient material0. Approximately 36 million reads were obtained across all FFPE samples and, after quality filtering and short read removal, >32 million reads were retained. The majority of reads (63%) mapped to the human or EBV genomes, with miRNAs constituting the predominant species of small RNAs identified (Figure 3). Analyses with miRDeep and miRExpress provided expression data for 984 and 847 known human and EBV miRNAs, respectively, each with greater than one count per million in at least two of the samples. Using EdgeR, 99 dysregulated miRNAs were identified in NPC tumor tissue versus non-neoplastic nasorespiratory control samples (Table 3). Approximately one-third (37) of these were of viral origin, all of which were up-regulated in the NPC tumor samples (Table 3).

**Table 3:** Small RNA-Seq analysis of NPC FFPE tissue

**Down-regulated**				
**miRNA**	**MiRDeep (logFC)**	**MiRExpress (logFC)**	**Family**	**NPC ref.**
hsa-let-7b-5p,hsa-let-7b	−1.23	−1.17	let-7/98/4458/4500	[36,50,51]
hsa-miR-100-5p,hsa-mir-100	−1.72	−1.57	miR-99ab/100	[36,50,52-54]
hsa-miR-1251,hsa-mir-1251	−5.69	−5.44	miR-1251	-
hsa-miR-1269a,hsa-mir-1269a	−6.44	-	miR-1269/1269b	-
hsa-miR-1269b,hsa-mir-1269b	−7.73	−6.14	miR-1269/1269b	-
hsa-miR-130a-3p,hsa-mir-130a	−2.82	−2.72	miR-130 ac/301ab/301b/301b-3p/454/721/4295/3666	[50,52,54]
hsa-miR-133a,hsa-mir-133a-1	−3.75	−3.63	miR-133abc	-
hsa-miR-133a,hsa-mir-133a-2	−3.75	−3.63	miR-133abc	-
hsa-miR-133b,hsa-mir-133b	−2.68	−2.74	miR-133abc	-
hsa-miR-136-5p,hsa-mir-136	−2.89	−2.77	miR-136	-
hsa-miR-139-5p,hsa-mir-139	−2.08	−2.04	miR-139-5p	[50,52,54]
hsa-miR-143-3p,hsa-mir-143	−1.64	−1.40	miR-143/1721/4770	[36,50,52,54]
hsa-miR-145-5p,hsa-mir-145	−2.77	−2.86	miR-145	[36]
hsa-miR-152,hsa-mir-152	−1.72	-	miR-148ab-3p/152	[50,52]
hsa-miR-187-3p,hsa-mir-187	−3.15	-	miR-187	[52]
hsa-miR-195-3p,hsa-mir-195	−2.37	−3.02	miR-15abc/16/16abc/195/322/424/497/1907	[50]
hsa-miR-195-5p,hsa-mir-195	−3.06	−2.29	miR-15abc/16/16abc/195/322/424/497/1907	[52]
hsa-miR-199a-5p,hsa-mir-199a-1	−2.46	−2.60	miR-199ab-5p	[55]
hsa-miR-199a-5p,hsa-mir-199a-2	−2.46	−2.60	miR-199ab-5p	[55]
hsa-mir-199b-5p,hsa-miR-199b	-	−2.10	miR-199ab-5p	[52]
hsa-miR-204-5p,hsa-mir-204	−4.68	−4.62	miR-204/204b/211	[52]
hsa-miR-214-3p,hsa-mir-214	−2.79	−2.83	miR-214/761/3619-5p	-
hsa-miR-3065-5p,hsa-mir-3065	−3.73	−3.60	miR-545/3065/3065-5p	-
hsa-miR-335-5p,hsa-mir-335	−2.71	−2.68	miR-335/335-5p	-
hsa-miR-376a-5p,hsa-mir-376a-1	−3.96	−3.75	miR-376abd/376b-3p	-
hsa-miR-376b-5p,hsa-mir-376b	−3.45	-	miR-376abd/376b-3p	-
hsa-miR-376c-5p,hsa-mir-376c	−3.45	-	miR-376c/741-5p	-
hsa-miR-4423-5p,hsa-mir-4423	−6.24	−6.51	miR-4423-5p	-
hsa-miR-450a-5p,hsa-mir-450a-1	-	−2.06	miR-450a/451a	-
hsa-miR-450a-5p,hsa-mir-450a-2	-	−2.05	miR-450a/451a	-
hsa-miR-4792,hsa-mir-4792	−1.53	-	miR-4792	-
hsa-miR-488-3p,hsa-mir-488	−4.86	−4.46	miR-488	-
hsa-miR-497-3p,hsa-mir-497	−3.63	-	miR-15abc/16/16abc/195/322/424/497/1907	[50,52]
hsa-miR-497-5p,hsa-mir-497	−2.53	−2.46	miR-15abc/16/16abc/195/322/424/497/1907	[50,52]
hsa-miR-504,hsa-mir-504	−3.31	-	miR-504/4725-5p	-
hsa-miR-539-5p,hsa-mir-539	−6.68	-	miR-300/381/539-3p	-
hsa-miR-542-3p,hsa-mir-542	−2.02	−1.96	miR-542-3p	-
hsa-miR-556-3p,hsa-mir-556	−5.18	-	miR-556-5p	-
hsa-miR-574-3p,hsa-mir-574	−1.32	−1.25	miR-574-3p	-
hsa-miR-585,hsa-mir-585	−2.51	-	miR-585	-
hsa-miR-874,hsa-mir-874	−1.68	−1.56	miR-874	-
hsa-miR-887,hsa-mir-887	−1.90	−1.99	miR-887	-
hsa-miR-891a,hsa-mir-891a	−7.14	−6.86	miR-891a	-
**Up-regulated**				
**miRNA**	**MiRDeep (logFC)**	**MiRExpress (logFC)**	**Family**	**NPC ref.**
hsa-mir-1268a,hsa-miR-1268a	-	2.89	miR-1268/1268b	-
hsa-mir-1268b,hsa-miR-1268b	-	2.59	miR-1268/1268b	-
hsa-miR-1303,hsa-mir-1303	1.61	1.62	miR-1303	-
hsa-miR-1304-3p,hsa-mir-1304	1.56	-	miR-1304	-
hsa-miR-1305,hsa-mir-1305	2.84	-	miR-1305	-
hsa-mir-15b-5p,hsa-miR-15b	-	1.09	miR-15abc/16/16abc/195/322/424/497/1907	[52]
hsa-miR-184,hsa-mir-184	4.84	5.22	miR-184	-
hsa-mir-21-3p,hsa-miR-21	-	1.41	miR-21/590-5p	-
hsa-mir-27a-3p,hsa-miR-27a	-	1.15	miR-27abc/27a-3p	-
hsa-miR-205-3p,hsa-mir-205	2.77	2.65	miR-205/205ab	[52]
hsa-miR-205-5p,hsa-mir-205	2.87	2.84	miR-205/205ab	[52]
hsa-miR-25-5p,hsa-mir-25	2.21	2.15	miR-25/32/92abc/363/363-3p/367	[52]
hsa-miR-4677-3p,hsa-mir-4677	1.52	1.74	miR-4677-3p	-
hsa-mir-4791,hsa-miR-4791	-	2.67	miR-3201/4791	-
hsa-mir-548n,hsa-miR-548n	-	3.79	miR-548abakhjiwy/548abcd-5p/559	-
hsa-miR-6510-3p,hsa-mir-6510	2.22	-	miR-6510-3p	-
hsa-miR-92a-3p,hsa-mir-92a-1	1.32	-	miR-25/32/92abc/363/363-3p/367	-
hsa-miR-92a-3p,hsa-mir-92a-2	1.28	-	miR-25/32/92abc/363/363-3p/367	-
hsa-mir-944,hsa-miR-944	-	1.47	miR-944	-
**EBV specific (All up-regulated)**				
**miRNA**	**MiRDeep (logFC)**	**MiRExpress (logFC)**		
ebv-miR-BART1-3p,ebv-mir-BART1	4.54	4.55		
ebv-miR-BART1-5p,ebv-mir-BART1	4.37	4.37		
ebv-miR-BART10-3p,ebv-mir-BART10	4.43	4.41		
ebv-miR-BART10-5p,ebv-mir-BART10	4.24	4.49		
ebv-miR-BART12,ebv-mir-BART12	3.52	3.47		
ebv-miR-BART13-3p,ebv-mir-BART13	2.64	2.55		
ebv-miR-BART13-5p,ebv-mir-BART13	4.29	4.32		
ebv-miR-BART14-3p,ebv-mir-BART14	3.63	3.61		
ebv-miR-BART14-5p,ebv-mir-BART14	3.78	3.83		
ebv-miR-BART15,ebv-mir-BART15	3.70	3.68		
ebv-miR-BART16,ebv-mir-BART16	3.52	3.53		
ebv-miR-BART17-3p,ebv-mir-BART17	4.45	4.44		
ebv-miR-BART17-5p,ebv-mir-BART17	4.81	4.72		
ebv-miR-BART18-3p,ebv-mir-BART18	3.73	3.28		
ebv-miR-BART18-5p,ebv-mir-BART18	4.44	4.44		
ebv-miR-BART19-3p,ebv-mir-BART19	3.51	3.47		
ebv-miR-BART19-5p,ebv-mir-BART19	4.58	-		
ebv-miR-BART2-5p,ebv-mir-BART2	3.79	3.72		
ebv-miR-BART20-3p,ebv-mir-BART20	3.41	3.39		
ebv-miR-BART20-5p,ebv-mir-BART20	4.55	4.62		
ebv-miR-BART21-3p,ebv-mir-BART21	2.79	2.71		
ebv-miR-BART21-5p,ebv-mir-BART21	3.51	3.50		
ebv-miR-BART22,ebv-mir-BART22	3.97	3.93		
ebv-miR-BART3-3p,ebv-mir-BART3	4.13	3.83		
ebv-miR-BART3-5p,ebv-mir-BART3	4.66	4.50		
ebv-miR-BART4-3p,ebv-mir-BART4	3.78	3.66		
ebv-miR-BART4-5p,ebv-mir-BART4	3.37	3.32		
ebv-miR-BART5-3p,ebv-mir-BART5	4.20	3.82		
ebv-miR-BART5-5p,ebv-mir-BART5	3.97	3.89		
ebv-miR-BART6-3p,ebv-mir-BART6	4.34	4.29		
ebv-miR-BART6-5p,ebv-mir-BART6	4.49	4.35		
ebv-miR-BART7-3p,ebv-mir-BART7	4.18	4.15		
ebv-miR-BART7-5p,ebv-mir-BART7	5.77	5.76		
ebv-miR-BART8-3p,ebv-mir-BART8	4.31	4.26		
ebv-miR-BART8-5p,ebv-mir-BART8	4.65	4.63		
ebv-miR-BART9-3p,ebv-mir-BART9	4.18	4.05		
ebv-miR-BART9-5p,ebv-mir-BART9	4.47	4.45		

Five FFPE samples previously analyzed by microarray were submitted for RNA-Seq and analyzed via both miRExpress and miRDeep tools. Significantly dysregulated miRNAs are listed in Table 3.

**Figure 4:**
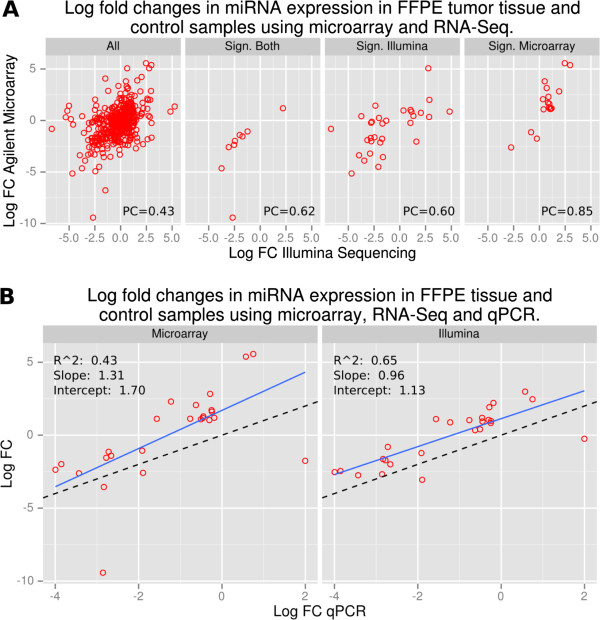
**Comparison of RNA-Seq and microarray in the analysis of FFPE NPC tissue. A)** The correlation of Fold Change (FC) values calculated by RNA-Seq and microarray analysis on FFPE NPC and control tissue are shown. The left panel includes miRNAs detected using both methods. The other panels show miRNAs identified as significantly dysregulated using both methods. Significantly dysregulated in only RNA-Seq (Sign. Illumina) and those identified using only microarray analysis (Sign. Microarray) are also shown. Pearson coefficient for each analysis is shown (PC). **B)** Comparison of FC values determined using qPCR and microarray analysis (first panel; Microarray) and qPCR and RNA-Seq (second panel; Illumina). Best simple linear regression line is shown (solid line) and the R^2^, y-intercept and slope from are denoted on each graph; Y = X is shown as a dotted line. Only miRNA’s included on the custom qPCR chip on the basis of their dysregulation in FFPE tissue using microarray analysis and/or RNA-Seq are shown.

MicroRNA expression levels obtained in microarray and RNA-Seq experiments were similar, with FC values obtained from the two methods showing a positive association (Pearson correlation 0.43; Figure 4A). Eighty-seven (87) human miRNA appeared to be dysregulated using either discovery method technology, with only 11 found in both analyses. There were 32 and 44 miRNAs identified independently by microarray and RNA-Seq, respectively (Figure 4A). We observed a strong positive correlation (Pearson correlation 0.85) between RNA-Seq and microarray FC values for miRNAs found to be significantly dysregulated in microarray analysis, and a weaker but still strong correlation for those identified as significantly dysregulated in the RNA-Seq analysis (Pearson correlation 0.60) (Figure 4A). All but three miRNAs identified as significantly dysregulated in the microarray analysis exhibited stronger up-regulation than in RNA-Seq, suggesting that cross-hybridization with closely related members of miRNA families could have inflated their intensities (Figure 4A). A similar effect, i.e. substantial differences in called differentially expressed miRNAs despite overall similarity in FC values, was recently reported when comparing the two platforms [56].

### Optimized protocols for qPCR from minimal sera volumes

We evaluated three different RNA purification kits using excess sera from healthy controls: the Qiagen miRNeasy, Qiagen miRNeasy serum/plasma, and Qiagen QIAamp Circulating Nucleic Acid kits. Using the manufacturers’ protocols, the Qiagen QIAamp kit was the most effective of these kits with yields between 10–27 ng/μl serum compared to 0.5-1.0 ng RNA/μl from the other kits tested. This provided at least 1 μg of total RNA from the volumes available, typically ~0.25 ml, which is an amount adequate for downstream applications such as RNA-Seq (≥1 μg total RNA usually required). The purified RNA exhibited 260/280 and 260/230 ratios of 1.6-1.9 and ~1.0, respectively. Analysis of recovered RNA using the Agilent BioAnalyzer 2100 showed 28S and 18S ribosomal RNA bands. However, the enrichment of small RNA species lower than 100 nt in size was not observed (data not shown). As was the case for FFPE samples, the RIN score (~3.0) suggested degradation of larger RNA species.

To verify miRNA expression levels in tumor tissue and provide a tool for the measurement of miRNA levels in sera, custom quantitative PCR (qPCR) plates were printed with primers for 40 selected miRNAs found to be significantly dysregulated in the FFPE tissue by microarray and RNA-Seq (Additional file 2). Given that only small volumes of sera were available, a preliminary examination of the effects of RNA concentration on qPCR was performed. Quantitative PCR experiments using both a small quantity of total RNA (30 ng), and an amount recommended by the manufacturers (250 ng) were conducted on RNA from both FFPE tissue and sera. A comparison of the expression values obtained using both concentrations revealed that, with one exception, no miRNAs displayed a FC difference greater than two when using these starting concentrations (Additional file 4). Given these results, 30 ng total RNA was used for subsequent experiments and a threshold of greater than two-fold dysregulation, in addition to significance value of *p* ≤ 0.05 was imposed when determining significantly dysregulated miRNA expression levels via qPCR.

### Expression levels determined using qPCR correlated with microarray and RNA-Seq levels

Quantitative PCR was performed on RNA from tumor and control tissue and expression levels showed good agreement with results from the microarray and RNA-Seq analyses. Of the 40 miRNAs included on the qPCR plate on the basis of their dysregulation in tissue, all were identified using qPCR. Using linear regression, ratios determined using RNA-Seq provided better correlation with qPCR results (R^2^ 0.65), while a number of outliers reduced the overall correlation of the microarray analysis with qPCR (R^2^ 0.43) (Figure 4B). Also evident were respective shifts of +0.70 and +0.13 in the y-intercept of the linear regression line for microarray and RNA-Seq analysis (Figure 4B). Shifts such as these have been observed when comparing ratios from qPCR to microarray or RNA-Seq ratios and they have been attributed to the use of external references in the normalization of qPCR results [56]. The use of external references for normalization makes qPCR sensitive to miRNA abundance when it varies between samples in relation to the external reference. For example, when miRNA abundance, as a proportion of total RNA, varies between samples and ribosomal RNA (which makes up the majority of total RNA) is used as the external reference. Microarray and RNA-Seq use normalization methods internal to the miRNA population and are not susceptible to this effect. In this analysis, SNORD was used to normalize qPCR results and thus positive shifts in the y-intercept could reflect the presence of less miRNA in tumor compared with control tissue.

### Expression levels of miRNAs found in FFPE were not reflected in the sera

**Figure 5:**
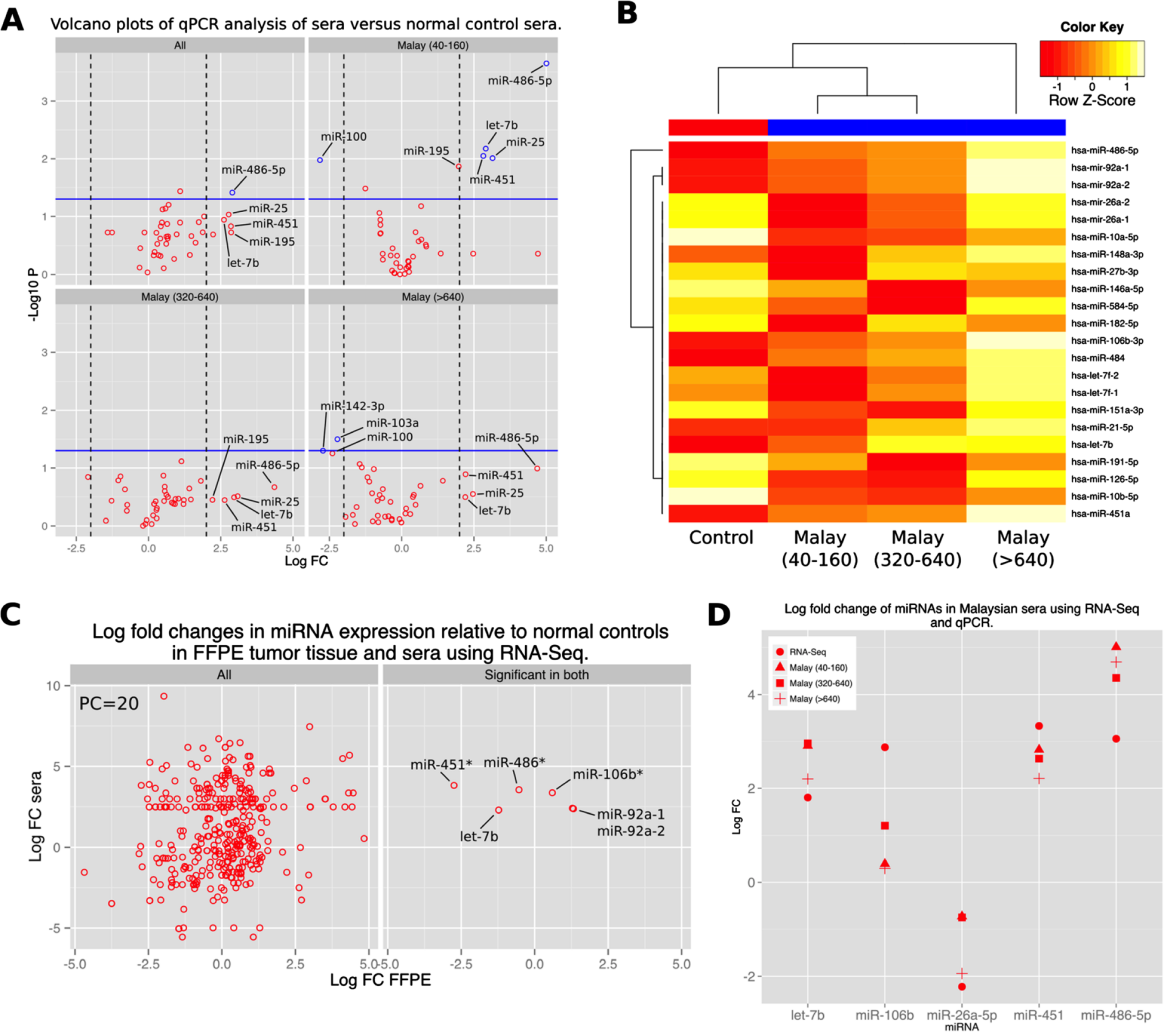
**Quantitative PCR and RNA-Seq analysis of serum sample from NPC patients.** Quantitative PCR was used to determine relative expression levels for 40 miRNAs in 40 sera samples comprised of 14 control samples and 26 test cases; 16 sera from Malaysian patients, 16 from German patients and eight from American patients (Malaysian, German, and American). **A)** Volcano plots of qPCR analysis of 40 sera samples. A single miRNA (miR-486-5p) was found to be significantly up-regulated when samples were analyzed as a whole (panel one; All). An analysis of Malaysian sera was also conducted incorporating anti-viral capsid antigen (VCA) IgG titers divided into four groups of four; a control group, low VCA titer group (second panel; Malaysian (40–160)), a medium VCA titer group (third panel; Malaysian (320–640)) and a high VCA group (fourth panel; Malaysian (> 640). Blue lines indicate a p-value of 0.05 and dashed lines show a four fold-change. Dysregulated miRNAs are shown in blue and labeled with the human mature miRNA identification number. **B)** Heatmap of significantly dysregulated miRNAs from RNA-Seq analysis of Malaysian sera. The heatmap was generated using the ‘heatmap.2′ function in the gplots R package and miRNA counts per million values were scaled across samples and colored to represent up-regulation (1.0) and down-regulations (−1.0). **C)** Comparison of FC values, generated using RNA-Seq, for miRNAs identified in FFPE and sera when compared to their respective controls. Left panel (All) shows comparison for all miRNAs identified in both analyses and the right panel (Significant in Both) for miRNAs identified as dysregulated in both tissues. Three miRNAs were identified as significantly up-regulated in sera but significantly down-regulated in FFPE tissue. Asterisk significant dysregulation in microarray analysis but not RNA-Seq. **D)** Comparison of FC values by qPCR and RNA-Seq in sera. Only miRNAs that were significantly dysregulated in RNA-Seq and were present on the custom qPCR chip are shown.

To quantify circulating miRNAs in NPC, 12 NPC positive sera were compared to sera from four healthy controls from a Malaysian sample set using qPCR. Test samples were from age (+/− 10 years), and sex (male) matched to the NPC patients, and each sample possessed associated serological analyses providing anti-viral capsid antigen (VCA) IgG titers [34]. When these samples were compared to healthy controls, only three significantly dysregulated miRNAs were identified; miR-486-5p and miR-451 were up-regulated, and miR-100 was down-regulated (fold change ≥ 2, *p* ≤ 0.05) (not shown). All these miRNAs had been significantly dysregulated in tumor tissue, using either RNA-Seq and/or microarray (Figure 2, Table 3). However, ratios for miR-451 and miR-486-5p showed that expression levels were inverted, indicating significant up-regulation in sera, but down-regulation in tumor tissue. Based on serology, the NPC cases were also analyzed as three individual groups: low (VCA IgG 40–160), medium (VCA IgG 320–640), and high (VCA IgG >640) antibody titers. In addition to miR-451, -486-5p, and −100, two additional miRNAs were identified as significantly up-regulated in the low titer group (miR-25 and let-7b) both of which were also identified as significantly dysregulated in tumor tissue, although let-7b had been identified as down-regulated (Figure 5A). Finally, additional sera from the U.S (five NPC sera and three matched healthy control sera) and Germany (10 NPC sera and six healthy control sera) were analyzed using the qPCR plates and the combined the results with those from the Malaysian sera. When combined, only miR-486-5p was identified as significantly up-regulated (Figure 5A).

Despite the qPCR plates being designed using miRNAs found to be dysregulated in NPC tumor tissue, few of these miRNAs were subsequently found in the sera of NPC cases. Therefore, RNA-Seq was used as an untargeted approach to profile the miRNAs in the Malaysian sera discussed above. Sixteen (16) Malaysian serum samples from individuals with histologically confirmed NPC were pooled into four groups corresponding to low, medium and high levels of EBV VCA titers as well as a control group with no detectable VCA (above). These four groups were then analyzed using the Illumina platform. Approximately 18 million reads were obtained from the four groups, with 33% of these reads (~5.7 million) mapped to miRNAs in miRBase (Figure 3). In total, 463 miRNAs were identified in these sera. Of these miRNAs, 416 were also identified in the RNA-Seq analysis of the FFPE tissue. Differential analysis of serum miRNA counts using EdgeR resulted in the identification of 20 miRNAs that were significantly dysregulated in serum from histologically confirmed NPC cases; 7 up-regulated and 13 down-regulated (Figure 5B, Table 4). A comparison of the relative expression levels of miRNAs in sera and FFPE showed little correlation (Pearson correlation 0.20; Figure 5C), indicating that the relative expression of miRNAs in NPC FFPE tissue was not reflected in sera from NPC cases. Six miRNAs that had been previously identified as dysregulated in sera using qPCR (above) were also identified using RNA-Seq and three of these, let-7b, miR-451a and miR-486, were shown to be significantly up-regulated in sera despite their significant down-regulation in tumor tissue (Figure 5C). The other 14 significantly dysregulated sera miRNAs had not been identified as dysregulated in tumors (Tables 3 and 4). Moreover, numerous miRNAs strongly dysregulated in tumor samples were absent from the sera (for example miR-205, miR-199a/b, and miR-139) (Tables 3 and 4). FC values of the miRNAs that were identified as significantly dysregulated in sera by RNA-Seq and were also on the custom qPCR plate used in analysis of sera showed similar values (Figure 5D). Finally, despite the significant dysregulation of 37 EBV miRNAs in tumor, no EBV miRNAs were found to be significantly dysregulated in sera.

**Table 4:** Small RNA-Seq analysis of sera of Malaysian cases

**Down-regulated**		
**miRNA**	**MiRDeep (logFC)**	**Family**
hsa-let-7f-1	−1.75	let-7/98/4458/4500
hsa-let-7f-2	−1.80	let-7/98/4458/4500
hsa-miR-10a	−2.71	miR-10abc/10a-5p
hsa-miR-10b	−3.04	miR-10abc/10a-5p
hsa-miR-126-5p	−2.43	miR-126-5p
hsa-miR-148a-3p	−1.42	miR-148ab-3p/152
hsa-miR-151a-3p	−2.37	miR-151-5p/151b
hsa-miR-182-5p	−2.20	miR-182
hsa-miR-21-5p	−0.99	miR-21
hsa-miR-26a-1	−2.22	miR-26ab/1297/4465
hsa-miR-26a-2	−2.23	miR-26ab/1297/4465
hsa-miR-27b-3p	−2.06	miR-27abc/27a-3p
hsa-miR-584-5p	−1.93	miR-584
**miRNA**	**MiRDeep (logFC)**	**Family**
hsa-let-7b	1.80	let-7/98/4458/4500
hsa-miR-106b-3p	2.87	miR-17/17-5p/20ab/20b-5p/93/106ab/427/518a-3p/519d
hsa-miR-451a	3.33	miR-451
hsa-miR-484	2.17	miR-344a-5p/484/3155/3155b
hsa-miR-486-5p	3.06	miR-486-5p/3107
hsa-miR-92a-1	1.91	miR-25/32/92abc/363/363-3p/36
hsa-miR-92a-2	1.88	miR-25/32/92abc/363/363-3p/36

Serum pools generated from individual samples used in qPCR analysis from were also subjected to small RNA-Seq. Table 4 shows the dysregulated miRNAs determined by the next generation sequencing.

### EBV miRNAs as potential NPC biomarkers

**Figure 6:**
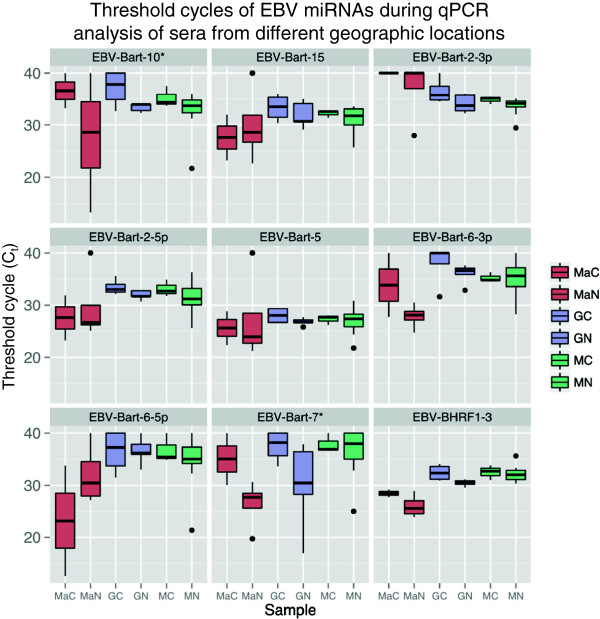
**Box and whisker plots of EBV miRNA expression levels in sera determined using qPCR.** Nine miRNAs were selected for qPCR based on their overexpression in FFPE NPC tissue. Box and whisker plots of the threshold cycle (Ct) values are shown for all miRNAs according to the geographical origin of the sera. MaC/MaN – Massachusetts control and test samples respectively (red); GC/GN – German control and test samples respectively (purple); and MC/MN – Malaysian control and test samples respectively (green). Boxes encompass the range between the upper and lower quartiles and whiskers extend to high/lowest values outside these bounds. Outliers, defined as greater/less than the upper/lower quartile by more than 1.5 times the interquartile range, are plotted as points.

**Figure 7:**
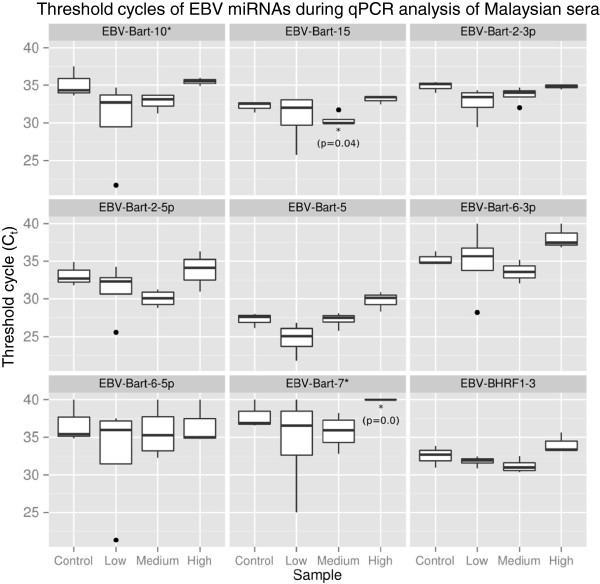
**Box and whisker plots of EBV miRNA expression levels in Malaysian sera determined using qPCR.** Box and whisker plots of the threshold cycle (Ct) values are shown for all miRNAs according to anti-viral capsid antigen (VCA) IgG titers. Low – IgG titers between 40–160; Medium – IgG titers between 320–640; and High – IgG titers greater than 640. Boxes encompass the range between the upper and lower quartiles and whiskers extend to high/lowest values outside these bounds. Outliers, defined as greater/less than the upper/lower quartile by more than 1.5 times the interquartile range, are plotted as points. Significantly dysregulated miRNAs are highlighted with asterisks.

Both microarray and RNA-Seq analysis identified significant up-regulation of EBV miRNAs in tumor tissue. Using RNA-Seq, 37 EBV miRNAs were identified as significantly up-regulated and microarray analysis of the same samples identified four up-regulated miRNAs (ebv-miR-BART4*, ebv-miR-BART6-5p, ebv-miR-BART6-3p, ebv-miR-BART5). Though close association between EBV and NPC suggests that EBV miRNAs could serve as NPC biomarkers, no EBV miRNAs were found to be significantly dysregulated in serum by RNA-Seq. While the average depth of RNA sequencing achieved on FFPE samples was approximately 3.5 million reads, the average depth for the serum samples was significantly lower at ~1.5 million. This difference may reflect the lower amount of miRNA contained in sera and, therefore, low abundance reads may not have been detected. To validate this finding, qPCR was also used to assay EBV miRNAs in sera. Nine (9) EBV specific primers were used to screen 40 total sera; 13 sera from healthy controls and 27 sera from NPC cases from three geographic locations (U.S., Germany, and Malaysia). While EBV miRNAs were detected in all sera, no miRNAs were significantly dysregulated when case sera were compared to control sera by qPCR. This is likely the result of the extensive variation observed in sera for these miRNAs (Figure 6). When the Malaysian sera were analyzed by VCA IgG titers strata (as described above), ebv-BART-15 was found to be significantly up-regulated in the mid-VCA (VCA IgG 320–640 titre) (FC = 3.51, *p* = 0.04) and Ebv-BART-7* in the high VCA sample (VCA IgG >640 titre) (FC = 1, p = <0.001) (Figure 7). Moreover, there appeared to be an inverse correlation between serology (VCA IgG) and EBV miRNA levels (Figure 7), i.e. the lowest NPC VCA titer group (IgG 40–160) displayed the highest positive FC in EBV miRNAs (FC 8–491) and the highest VCA titer group (>1280) showed the lowest FC (0.39-1.02) (values < 1.0 considered a negative FC). Overall, EBV levels showed great variability, even within sera collected from a single sample population (e.g., Malaysian sera) and even when these populations were stratified based on the strength of the VCA titer. More specifically, the higher the Ct for a particular miRNA, the lower the observed VCA titer (Figure 7). In this regard, we found that lowest NPC VCA titer group (IgG 40–160) displayed the highest positive FC in EBV miRNAs (FC 8–491) and the highest VCA titer group (>1280) showed the lowest FC (0.39-1.02) (values < 1.0 considered a negative FC) (not shown).

## Conclusions

Nasopharyngeal carcinoma (NPC) is a squamous cell carcinoma of the head and neck, unique for its diverse geographical clustering and its strong association with Epstein Barr virus (EBV). Early detection of NPC is difficult due the location of the tumor (deep in the nasopharynx) and the lack of obvious clinical signs in the early stages [57]. While there is an excellent response to multimodal treatment (chemoradiation therapy) when NPC is detected early, the prognosis after a late diagnosis of NPC is dismal. Hence, there is an urgent need for an accessible biomarker for the early detection of NPC. The aberrant expression of miRNAs in carcinogenesis has propelled these small non-coding RNAs to the forefront of recent cancer biomarker research [58]. One advantage of miRNAs is their stability in biofluids, including sera, plasma, urine, and saliva despite harsh conditions such as high temperatures, extreme pH values, repeated freeze–thaws, and long-term storage [59]. Moreover, miRNAs generally survive intact in tissues that have been fixed in formalin and embedded in paraffin (FFPE) for years [48].

With its extensive interaction with the host periphery, we hypothesized that NPC primary tumors would secrete miRNAs into the blood stream as shown for other solid tumors (e.g., metastatic breast, colon, and prostate cancers) [3]. Accordingly, we sought to test different methods to identify signatures of miRNAs in NPC FFPE tumor tissue versus non-neoplastic nasorespiratory control tissue and initiated this methods testing by interrogating FFPE using two approaches. The first approach was “targeted” method, where a platform of known miRNAs were surveyed in FFPE samples by microarray using miRBase 16. The second approach was “untargeted” method, where a high throughput analysis of all small RNA species in FFPE, including yet to be discovered miRNAs, were identified by RNA-Seq. Using these two approaches similar miRNA profiles were identified by microarray and RNA-Seq, with significantly dysregulated miRNAs then verified in both FFPE and sera by qPCR.

With the exception of three miRNAs (−106b and -92a-1/2), we found that miRNA expression levels in NPC FFPE tissue were not necessarily reflected in miRNA expression profiles in sera from NPC cases, though both miRNAs expression profiles strongly associating with NPC. Moreover, three of the overlapping miRNAs found in both sera and tumor tissue were inversely correlated (Figure 5A and 5C). Differing miRNA dysregulation profiles for tumor tissue and sera have been described for other cancers, including breast cancer, where several miRNAs have been shown to have an inverse expression in the tumor compared to sera [60]. As such, we add to the literature on the methods for measuring miRNAs yet another example of different miRNA profiles in tissue and serum for the same cancer, with both signatures strongly associated with the malignancy. The finding of divergent expression profiles in sera and tumor tissue is especially intriguing for NPC given the extensive interaction of this solid tumor with the host [3]. Peripheral blood and saliva from NPC patients often contain tumor-derived metabolites, including cytokines, non-cytokine tumor proteins, and viral nucleic acids, as well as EBV antibodies and antigens [3].

Among the more persuasive hypotheses to explain the divergent miRNA expression profiles between tissue and sera is that the majority of human extracellular miRNAs are encapsulated in microvesicles called ‘exosomes’ that can be isolated from serum [61]. In particular, NPC-related miRNAs, including EBV miRNAs, circulate in the plasma within exosomes and play important roles in promoting angiogenesis, cell proliferation, tumor-cell invasion and immune evasion [3,6,62]. However, recent reports demonstrate the presence of the EBV miRNA BART17 in plasma in the non-exosomal fraction [63]. Moreover, miRNAs isolated from the non-exosomal fraction of both plasma and sera have been found to be associated with Argonaute 2, a key effector enzyme of miRNA-mediated silencing [64]. Since the aim of our analysis was to detect as many candidate miRNAs as possible in sera, we isolated RNA from whole serum rather than exosomes or exosome-depleted serum. Using this approach, we ensured that most of the exosomal and non-exosomal miRNAs were available for detection [61]. In addition, the RNA isolation protocol that we used has been used by others to recover miRNAs, not only from exosomal enriched serum fractions, but from whole serum [65]. While the observed difference in the presence of miRNAs between primary tissue and sera cannot be explained by the sequestering of miRNAs in exosomes, it could be that there is a selective secretion of a particular set of miRNAs in exosomes derived from the NPC cancer cell and/or cells present in the tumor microenvironment.

A second objective of the study was to assess different methods that could be utilized for biomarker discovery of c-miRNAs for NPC. As such, we compared miRNA expression profiles in FFPE by parallel technologies: a ‘targeted’ discovery method represented by microarrays, where known miRNAs are surveyed by a release 16 human miRNAs (miRBase r16), and an ‘untargeted’ discovery method, where all miRNA copies present in a sample (including unknown miRNAs) are surveyed by small RNA sequencing on the Illumina platform. When utilized in FFPE and sera, both platforms enabled us to narrow the candidate miRNA signature to ~1-5% of the known mature human miRNAs: e.g., RNA-Seq analysis of FFPE and serum identified 99 and 20 dysregulated miRNAs associated with NPC, respectively, from the more than 2,200 human mature miRNAs in miRBase Release 19.0. Hence, these platforms significantly reduced the number of ‘candidate’ miRNAs for an NPC signature and allowed the use of a more cost effective method (qPCR) to verify miRNAs in sera. Among the more important points that arose from our study of different miRNA discovers methods using different sample types is that due to the low abundance of miRNAs in sera and the significantly lower average reads obtained by RNA-seq in sera samples versus FFPE samples (~1.5 million versus 3.5 million, respectively), future studies should increase the sequencing depth when sera is used as the sample matrix in order to detect low abundance miRNAs.

While successful prognostic miRNA profiling has been demonstrated for NPC using targeted discovery platforms (microarray) in FFPE [36], this study is the first to assess available methods to identify NPC biomarkers using both targeted and untargeted miRNA discovery technologies on different sample types (Figure 1) [66]. We found miRNA profiles were consistent between the two microarray (targeted) and RNA-Seq (untargeted) when these two discovery technologies are applied to the same sample matrix: e.g. microarray versus RNA-Seq applied to FFPE tissue. A benefit of the untargeted biomarker discovery technology (RNA-Seq) was the identification of novel (i.e., unknown) miRNAs associated with NPC. Approximately 20 novel miRNA candidates were identified in the study and are currently the objective of future studies and verification by our group. These novel miRNAs may indeed prove valuable as potential biomarkers for NPC, with further experimentation (including PCR validation). However, as mentioned above, when the same discovery technologies were applied to a different sample matrices (serum), there was little overlap in dysregulated miRNAs associated between the two NPC types, suggesting that sera and tissue may have different miRNA profiles for NPC. The absence of overlapping miRNAs between sera and tissue as determined by both RNA-Seq and microarray was verified by qPCR step.

Whereas RNA-Seq has been extensively utilized on FFPE, much less information has been reported on RNA-Seq of sera or plasma [19]. The average reads obtained per serum sample for both serum and plasma as well as their mapping are shown in Figure 3. From each individual serum sample, we obtained ~1 million miRNA reads (~45% of the total mapped reads). In FFPE samples, an average of > 2.5 million miRNA reads per sample (>60% of total mapped reads) were obtained. Other significant reads obtained from both samples had no annotation, and a small percentage from FFPE contained reads mapped to small nuclear RNA, protein coding, and other (long non-coding RNA, antisense RNA, and vault RNA). Although both qPCR and RNA-Seq of sera provided no significantly dysregulated EBV miRNAs, qPCR clearly detected the presence of EBV miRNAs in NPC case compared to control sera. Conversely, the raw copy counts for EBV miRNAs in RNA-Seq were low or non-existent; suggesting that the sequencing depth obtained in RNA-Seq of sera was not sufficient to identify low abundance miRNAs. As mentioned previous, an important result of this manuscript may be the need to increase the depth of sequencing for miRNA when examining sera.

As an EBV associated malignancy, the expression of EBV immunogenic proteins and antibodies in both tumor tissue and blood were expected, and have been found to be indicative of an immune response against these carcinogenic proteins [62]. Hence, we anticipated the occurrence of EBV miRNAs in sera in similar patterns as found in previous studies. For example, EBV derived miRNAs (such as BART miRNAs) have been detected in the sera of NPC patients and have been considered potential candidates for circulating NPC biomarkers [6,63]. Although 37 dysregulated EBV miRNAs were identified in FFPE by RNA-Seq, we were unable to discern a consistent and significant EBV miRNA signature in the serum samples associated with NPC. Most notably, there was a marked variability in miRNA levels in sera across different geographic locations. Even when limited to a single geographic location, such as sera from Malaysia, wide variation was observed in EBV miRNA expression levels and significant differences between miRNA in cases compared to controls could not be identified, although some EBV miRNA expression levels did seem to be inversely correlated with VCA titer.

In summary, this comparative analysis of the available methods for the discovery of biomarkers in different sample types revealed important information concerning circulating miRNAs for NPC. First, it showed that optimized extraction protocols could produce sufficient RNA from FFPE and sera for miRNA discovery and verification. Second, our study showed the marked reproducibility between the two different miRNA discovery platforms when applied to FPPE, i.e., both targeted (microarray) and untargeted (RNA-Seq) discovery platforms provided comparable miRNA expression profiles when applied to FFPE tumor and healthy tissue controls, although statistical methods for determining significance provide different sets of significantly differentiated miRNAs. Third, c-miRNA expression profiles in the sera of NPC cases differed from the miRNA expression profiles in tumor FFPE, which may require future studies to increase the sequencing depth when sera is used as the sample matrix in order to detect low abundance miRNAs. Finally, when there was an overlap of miRNAs between FFPE and sera, the miRNAs tended to be inversely regulated. The latter two findings were unexpected given the assumption that biomarker discovery should start from the primary tumor to develop candidate biomarkers which could be verified in the sera. Finally, we concluded that the untargeted RNA-Seq approach applied to sera is the most informative method for discovering circulating miRNAs associated with NPC and possibly other cancers as well given its untargeted nature.

## Abbreviations

EBV: Epstein Barr virus; FC: Fold change; FFPE: Formalin fixed paraffin embedded; miRNA: MicroRNA; NPC: Nasopharyngeal carcinoma; NGS: Next generation sequencing; qPCR: Quantitative real-time PCR; RNA-seq: Small RNA-sequencing/RNA sequencing; TNM: Tumor, node, and metastasis staging; VCA: Anti-viral capsid antigen.

## Competing interests

The authors declare that they have no competing interests.

## Authors’ contributions

JLP, GR, YF performed the sample isolation and experiments including microarray and qPCR. JPM performed RNA-Seq analysis and related Figures. PL, SE, EM, SH, NS, and JMB supplied sample/patient cohorts, pathology and related figures. JLP, GR, JMB, and JPM conceived and designed the experiments. JLP, GR, YF, PL, SE, PJB, JMB, and JPM assisted in coordination and manuscript preparation. All authors reviewed and approved the final manuscript.

## Supplementary Material

Additional file 1 **Representative images of non-keratinizing nasopharyngeal carcinoma (NPC) **[40]** and non-neoplastic tissue.** Differentiated NPC (A) H&E (20X); (B) tumor cells with well-demarcated cell borders and abundant cytoplasm, H&E (100X oil immersion). Undifferentiated NPC [40]: (C) syncytial groups of tumor cells with associated lymphocytes (*short arrows*) and plasma cells (*arrowheads*), H&E (20X), (inset) in situ hybridization for EBV (EBER) positive in tumor cells; (D) tumor cells with vesicular nuclear chromatin (*long arrows*) and prominent nucleoli (*arrowheads*); mature lymphocytes (*short arrows*) are present in the background, H&E (100Xoil immersion). Non-neoplastic nasopharyngeal mucosa: (E,F) normal ciliated (arrow) nasorespiratory surface epithelium, H&E (10x, 40X respectively). Click here for file

Additional file 2 **miRNAs and controls utilized on custom printed PCR plates (SABiosciences) for verification in serum via qPCR. **The top 40 dysregulated miRNAs were selected based on the analysis of microarray data from FFPE. Click here for file

Additional file 3 **miRNA expression profiles from eight samples by Agilent microarray (miR release v. 16).** Eight FFPE samples (four NPC, four control) were analyzed using an unpaired *t*-test. miRNAs with significant up or down regulations (fold change > 2.0 and *p* < 0.05) are listed. Only miRNAs where ≥2 samples had raw values >20.0 are presented. Click here for file

Additional file 4 **Scatter plot of miRNA qPCR findings from various cDNA preparations of the same sample.** Scatter plot of miRNA qPCR findings from cDNA prepared from 30 ng total RNA (y-axis) and 250 ng total RNA(x-axis) derived from FFPE (Panel A) and Caucasian sera (Panel B). Diagonal lines indicate 2-fold threshold boundaries. Click here for file
