# Supplementary material for: Methods and matrices: approaches to identifying miRNAs for Nasopharyngeal carcinoma
Source: J Transl Med. 2014 Jan 6;12:3. doi: 10.1186/1479-5876-12-3 (PMC3895762; doi:10.1186/1479-5876-12-3)

Additional File 1. Representative images of non-keratinizing nasopharyngeal carcinoma (NPC) and non-neoplastic tissue. Differentiated NPC (A) H&E (20X); (B) tumor cells with well-demarcated cell borders and abundant cytoplasm, H&E (100X oil immersion). Undifferentiated NPC: (C) syncytial groups of tumor cells with associated lymphocytes (*short arrows*) and plasma cells (*arrowheads*), H&E (20X), (inset) in situ hybridization for EBV (EBER) positive in tumor cells; (D) tumor cells with vesicular nuclear chromatin (*long arrows*) and prominent nucleoli (*arrowheads*); mature lymphocytes (*short arrows*) are present in the background, H&E (100Xoil immersion). Non-neoplastic nasopharyngeal mucosa: (E,F) normal ciliated (arrow) nasorespiratory surface epithelium, H&E (10x, 40X respectively).

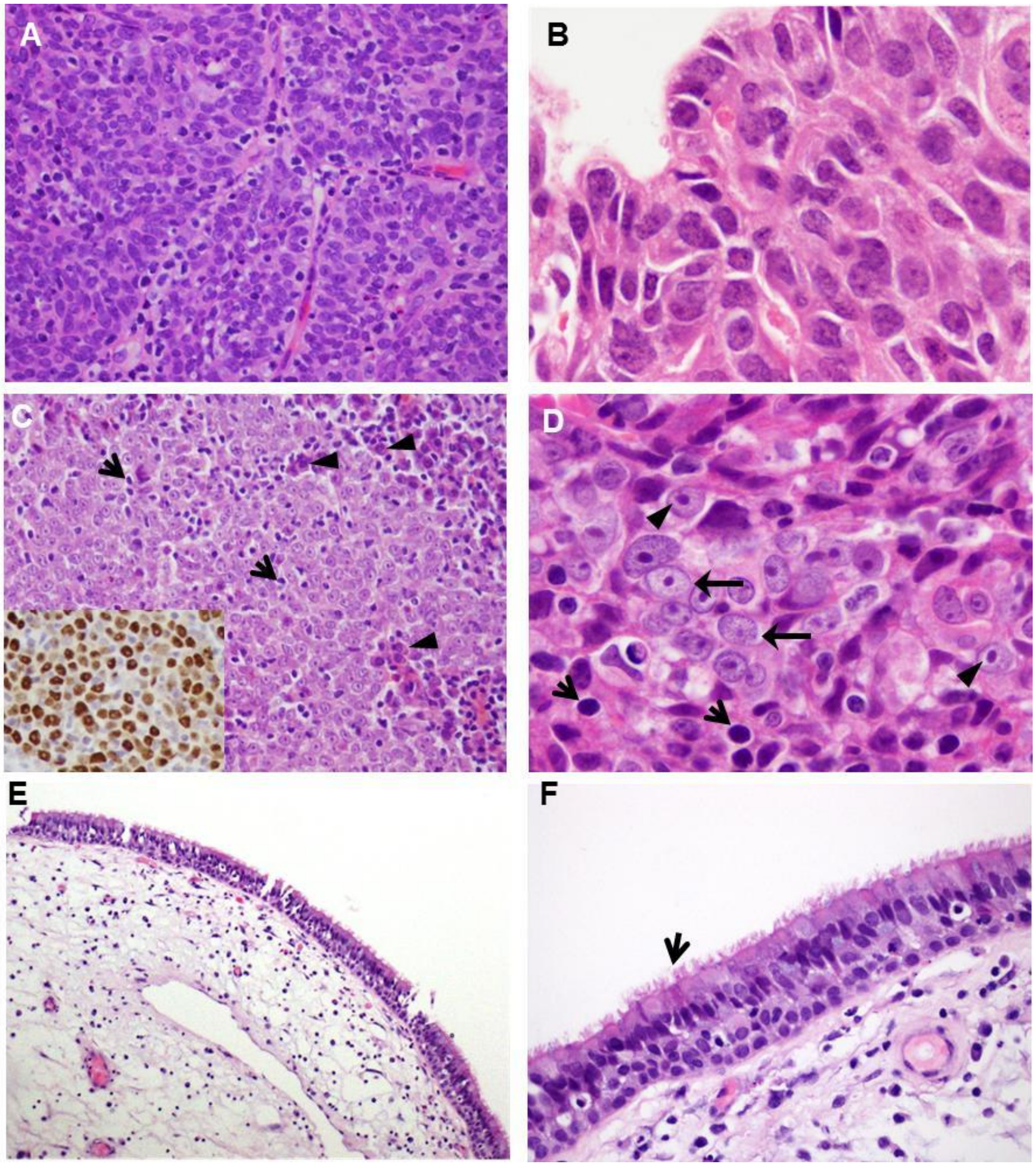

Supplement: Additional file 1 — Representative images of non-keratinizing nasopharyngeal carcinoma (NPC) [40] and non-neoplastic tissue. Differentiated NPC (A) H&E (20X); (B) tumor cells with well-demarcated cell borders and abundant cytoplasm, H&E (100X oil immersion). Undifferentiated NPC [40]: (C) syncytial groups of tumor cells with associated lymphocytes (short arrows) and plasma cells (arrowheads), H&E (20X), (inset) in situ hybridization for EBV (EBER) positive in tumor cells; (D) tumor cells with vesicular nuclear chromatin (long arrows) and prominent nucleoli (arrowheads); mature lymphocytes (short arrows) are present in the background, H&E (100Xoil immersion). Non-neoplastic nasopharyngeal mucosa: (E,F) normal ciliated (arrow) nasorespiratory surface epithelium, H&E (10x, 40X respectively). [file 1479-5876-12-3-S1.pdf]
