# Supplementary material for: Methods and matrices: approaches to identifying miRNAs for Nasopharyngeal carcinoma
Source: J Transl Med. 2014 Jan 6;12:3. doi: 10.1186/1479-5876-12-3 (PMC3895762; doi:10.1186/1479-5876-12-3)
Supplement: Additional file 2 — miRNAs and controls utilized on custom printed PCR plates (SABiosciences) for verification in serum via qPCR. The top 40 dysregulated miRNAs were selected based on the analysis of microarray data from FFPE. [file 1479-5876-12-3-S2.pdf]

Additional File 2. miRNAs and controls utilized on custom printed PCR plates (SABiosciences) for verification in serum via qPCR. The top 40 dysregulated miRNAs were selected based on the analysis of microarray data from FFPE.

Mature miRNA Sanger ID	Mature miRNA Accession
hsa-let-7b	MIMAT0000063
hsa-miR-195	MIMAT0000461
hsa-miR-1287	MIMAT0005878
hsa-miR-25	MIMAT0000081
hsa-miR-17	MIMAT0000070
hsa-miR-93	MIMAT0000093
hsa-miR-106b	MIMAT0000680
hsa-miR-107	MIMAT0000104
hsa-miR-223	MIMAT0000280
hsa-miR-196b	MIMAT0001080
hsa-miR-497	MIMAT0002820
hsa-miR-1275	MIMAT0005929
hsa-miR-130b	MIMAT0000691
hsa-miR-133b	MIMAT0000770
hsa-miR-3138	MIMAT0015006
hsa-miR-3651	MIMAT0018071
hsa-miR-20a	MIMAT0000075
hsa-miR-1260b	MIMAT0015041
hsa-miR-3663-3p	MIMAT0018085
hsa-miR-486-5p	MIMAT0002177
hsa-miR-451	MIMAT0001631
hsa-miR-221	MIMAT0000278
hsa-miR-125b	MIMAT0000423
hsa-miR-100	MIMAT0000098
hsa-miR-15b	MIMAT0000417
hsa-miR-199b-5p	MIMAT0000263
hsa-miR-149	MIMAT0000450
hsa-miR-720	MIMAT0005954
hsa-miR-1260	MIMAT0005911
hsa-miR-103a	MIMAT0000101
hsa-miR-141	MIMAT0000432
hsa-miR-199a-3p	MIMAT0000232
hsa-miR-142-3p	MIMAT0000434
hsa-miR-29c	MIMAT0000681
hsa-miR-26a	MIMAT0000082
hsa-miR-30e	MIMAT0000692
hsa-miR-143	MIMAT0000435
hsa-miR-203	MIMAT0000264
hsa-miR-34c-5p	MIMAT0000686
hsa-miR-99a	MIMAT0000097
SNORD61	miScript PCR Control snoRNA
SNORD72	miScript PCR Control snoRNA
SNORD95	miScript PCR Control snoRNA
SNORD96A	miScript PCR Control snoRNA
miRTC	Reverse Transcription Control
miRTC	Reverse Transcription Control
PPC	Positive PCR Control
PPC	Positive PCR Control
