# Supplementary material for: Methods and matrices: approaches to identifying miRNAs for Nasopharyngeal carcinoma
Source: J Transl Med. 2014 Jan 6;12:3. doi: 10.1186/1479-5876-12-3 (PMC3895762; doi:10.1186/1479-5876-12-3)
Supplement: Additional file 3 — miRNA expression profiles from eight samples by Agilent microarray (miR release v. 16). Eight FFPE samples (four NPC, four control) were analyzed using an unpaired t-test. miRNAs with significant up or down regulations (fold change > 2.0 and p < 0.05) are listed. Only miRNAs where ≥2 samples had raw values >20.0 are presented. [file 1479-5876-12-3-S3.pdf]

Additional file 3. miRNA expression profiles from eight samples by Agilent microarray (miR release v. 16). Eight FFPE samples (four NPC, four control) were analyzed using an unpaired *t*-test. miRNAs with significant up or down regulations (fold change>2.0 and p<0.05) are listed. Only miRNAs where ≥ 2 samples had raw values > 20.0 are presented .

miRNA	p-Value	Fold Change (Abs)
Down Regulated		
hsa-miR-133b	0.014	688.8
hsa-miR-451	0.025	6.1
hsa-miR-195	0.002	6.0
hsa-miR-497	0.009	5.2
hsa-miR-199b-5p	0.040	4.0
hsa-miR-486-5p	0.022	3.9
hsa-miR-1275	0.012	3.4
hsa-miR-100	0.037	3.0
hsa-miR-199a-3p	0.046	2.7
hsa-miR-3663-3p	0.019	2.5
hsa-miR-3138	0.016	2.3
hsa-miR-125b	0.027	2.2
hsa-let-7b	0.001	2.1
Up-Regulated		
hsa-miR-196b	0.008	47.3
hsa-miR-203	0.013	41.6
hsa-miR-18a	0.037	14.0
hsa-miR-17*	0.037	8.9
hsa-miR-149	0.040	7.1
hsa-miR-141	0.046	4.9
hsa-miR-221	0.025	4.2
hsa-miR-93	0.003	3.3
hsa-miR-3651	0.002	3.1
hsa-miR-1274a	0.007	3.0
hsa-miR-17	0.003	2.4
hsa-miR-1274b	0.018	2.4
hsa-miR-25	0.002	2.3
hsa-miR-1260	0.041	2.3
hsa-miR-20a	0.019	2.2
hsa-miR-130b	0.014	2.2
hsa-miR-106b	0.006	2.1
hsa-miR-15b	0.039	2.0
EBV Specific (All up-regulated)		
ebv-miR-BART4*	0.024	100.0
ebv-miR-BART6-5p	0.043	99.9
ebv-miR-BART6-3p	0.041	92.8
ebv-miR-BART5	0.027	69.0
