# Supplementary material for: Methods and matrices: approaches to identifying miRNAs for Nasopharyngeal carcinoma
Source: J Transl Med. 2014 Jan 6;12:3. doi: 10.1186/1479-5876-12-3 (PMC3895762; doi:10.1186/1479-5876-12-3)

Additional File 4. Scatter plot of miRNA qPCR findings from cDNA prepared from 30 ng total RNA (y-axis) and 250 ng total RNA(x-axis) derived from FFPE (Panel A) and Caucasian sera (Panel B). Diagonal lines indicate 2-fold threshold boundaries.

A.

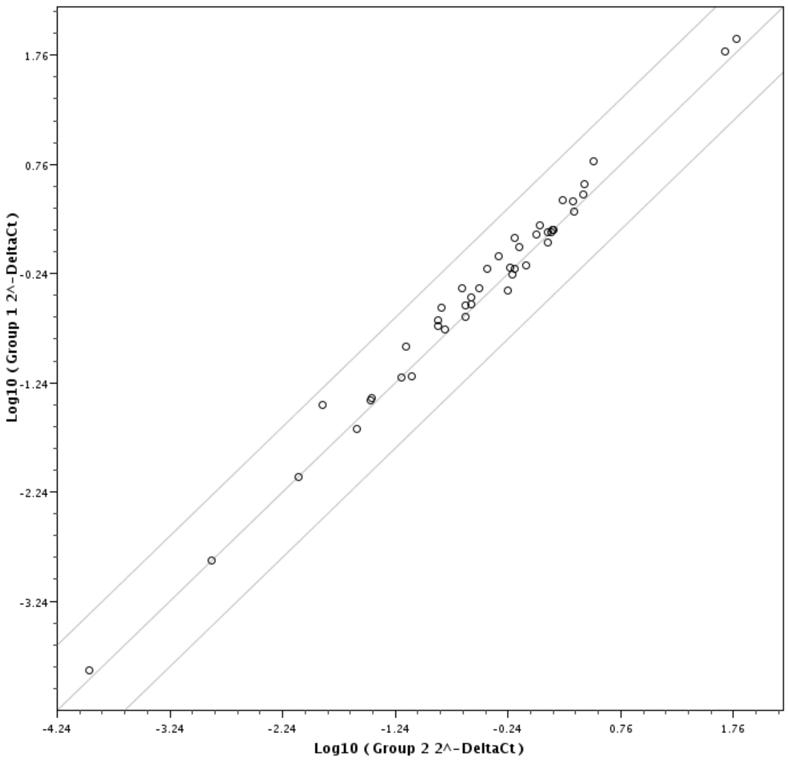

B.

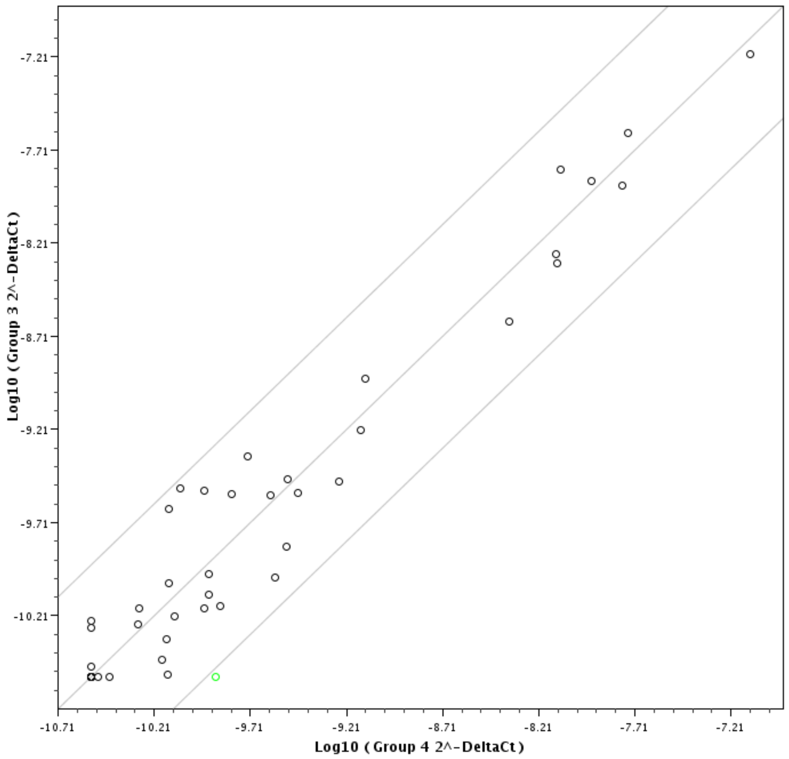

Supplement: Additional file 4 — Scatter plot of miRNA qPCR findings from various cDNA preparations of the same sample. Scatter plot of miRNA qPCR findings from cDNA prepared from 30 ng total RNA (y-axis) and 250 ng total RNA(x-axis) derived from FFPE (Panel A) and Caucasian sera (Panel B). Diagonal lines indicate 2-fold threshold boundaries. [file 1479-5876-12-3-S4.pdf]
